# Dual Erb B Inhibition in Oesophago-gastric Cancer (DEBIOC): A phase I dose escalating safety study and randomised dose expansion of AZD8931 in combination with oxaliplatin and capecitabine chemotherapy in patients with oesophagogastric adenocarcinoma

**DOI:** 10.1016/j.ejca.2019.10.010

**Published:** 2020-01

**Authors:** Anne Thomas, Pradeep S. Virdee, Martin Eatock, Simon R. Lord, Stephen Falk, D. Alan Anthoney, Richard C. Turkington, Matthew Goff, Leena Elhussein, Linda Collins, Sharon Love, Joanna Moschandreas, Mark R. Middleton

**Affiliations:** a University of Leicester, Leicester, UK; b Centre for Statistics in Medicine, University of Oxford, Oxford, UK; c Belfast City Hospital, Belfast, UK; d University of Oxford, Oxford, UK; e Bristol Haematology & Oncology Centre, Bristol, UK; f St. James University Hospital, Leeds, UK; g Centre for Cancer Research and Cell Biology, Queens University Belfast, Belfast, UK; h Oncology Clinical Trials Office, University of Oxford, Oxford, UK; i NIHR Oxford Biomedical Research Centre, UK

**Keywords:** Oesophagogastric cancer, Dual erbB inhibitor, AZD8931

## Abstract

**Background:**

AZD8931 has equipotent activity against epidermal growth factor receptor, erbB2, and erbB3. Primary objectives were to determine the recommended phase II dose (RP2D) of AZD8931 + chemotherapy, and subsequently assess safety/preliminary clinical activity in patients with operable oesophagogastric cancer (OGC).

**Methods:**

AZD8931 (20 mg, 40 mg or 60 mg bd) was given with Xelox (oxaliplatin + capecitabine) for eight 21-day cycles, continuously or with intermittent schedule (4 days on/3 off every week; 14 days on/7 off, per cycle) in a rolling-six design. Subsequently, patients with OGC were randomised 2:1 to AZD8931 + Xelox at RP2D or Xelox only for two cycles, followed by radical oesophagogastric surgery. Secondary outcomes were safety, complete resection (R0) rate, six-month progression-free survival (PFS) and overall survival.

**Results:**

During escalation, four dose-limiting toxicities were observed among 24 patients: skin rash (1) and failure to deliver 100% of Xelox because of treatment-associated grade III-IV adverse events (AEs) (3: diarrhoea and vomiting; vomiting; fatigue). Serious adverse events (SAE) occurred in 15 of 24 (63%) patients. RP2D was 20-mg bd with the 4/3 schedule. In the expansion phase, 2 of 20 (10%) patients in the Xelox + AZD8931 group and 5/10 (50%) patients in the Xelox group had grade III–IV AEs. Six-month PFS was 85% (90% CI: 66%–94%) in Xelox + AZD8931 and 100% in Xelox alone. Seven deaths (35%) occurred with Xelox + AZD8931 and one (10%) with Xelox. R0 rate was 45% (9/20) with Xelox + AZD8931 and 90% (9/10) with Xelox-alone (*P* = 0.024).

**Conclusion:**

Xelox + AZD8931 (20 mg bd 4/3 days) has an acceptable safety profile administered as neoadjuvant therapy in operable patients with OGC. (Trial registration: EudraCT 2011-003169-13, ISRCTN-68093791).

## Highlights

• First study to assess safety and tolerability of the novel pan erbB inhibitor AZD8931 in oesophagogastric cancer (OGC).
• Recommended phase II dose schedule determined to be 20-mg bd for 4 days on/3 days off, per cycle.
• Feasibility of adding novel targeted therapies to standard-of-care for oesophago-gastric cancer is confirmed.

## 1 Introduction

In the UK, gastric and oesophageal cancers account for approximately 16,000 cases per annum with mortality approaching 13,000 cases per annum [1]. In the western world, oesophageal adenocarcinoma incidence rates have increased markedly over the last 30 years [2] with the majority of patients presenting with advanced disease [3]. The UK national oesophagogastric cancer (OGC) audit (2018) determined that only 38.6% of patients were treated with curative intent, with a 5-year overall survival (OS) of 15% for oesophageal and 19% for gastric cancers [4]. There is, therefore, an urgent need to develop more effective therapies.

Patients with operable OGC may be treated with neoadjuvant chemotherapy [5], perioperative chemotherapy [[6], [7], [8]], or preoperative chemoradiotherapy [9]. The choice of therapy depends on the location of the tumour, the histology (squamous versus adenocarcinoma), the patient's performance status, and their comorbidity. Several other strategies, including treatment intensification [10] and anti-angiogenic therapies [11], have failed to demonstrate additional benefit in operable patients. Trastuzumab has demonstrated activity in patients with advanced human epidermal growth factor receptor-2 (EGFR) (HER2/erbB2) positive OGC [12,13], and results from neoadjuvant studies using this in addition to chemotherapy are awaited [14,15].

Whilst HER2 is an established target in OGC, further molecular subclassifications include overexpression of EGFR (HER1/erbB1) and heterodimeric activation of HER2 via erbB3 (HER3) [16,17], advocating extension of therapeutic targeting encompassing the wider EGFR family. AZD8931 is a novel small-molecule inhibitor, which has equipotent activity against signalling by EGFR, erbB2, and erbB3. In preclinical models, AZD8931 has greater anti-cancer activity than other EGFR inhibitors, such as gefitinib and lapatinib, which have narrower spectrums of erbB receptor inhibition [18]. AZD8931 combined with chemotherapy may, therefore, have activity in a wider group of patients with OGC (and other solid tumour) than for those just exhibiting highly HER2 amplified disease.

The Dual Erb B Inhibition in Oesophago-gastric Cancer (DEBIOC) study sought to establish the maximum tolerated dose (MTD) of AZD8931 in combination with oxaliplatin and capecitabine (Xelox) in patients with OGC (dose escalation phase). After establishment of the recommended phase II dose (RP2D), the dose expansion phase aimed to give preliminary efficacy assessments and further investigate the safety and tolerability of AZD8931 in combination with Xelox.

## 2 Materials and methods

The DEBIOC study was conducted in accordance with the International Conference of Harmonisation of Good Clinical Practice and the Declaration of Helsinki. Ethical approval was provided by a research ethics committee (12/SC/0090).

### 2.1 Patients

For dose escalation, eligible participants were chemonaive with inoperable metastatic OGC (measurable by RECIST 1.1), aged ≥18 years, had World Health Organisation performance status of 0 or 1 and adequate haematological, renal, hepatic, respiratory, and cardiac function. For dose expansion, eligible patients had histologically confirmed operable adenocarcinoma of the oesophagus or gastrooesophageal junction, including Siewert type I and II gastrooesophageal junction cancers, and were deemed suitable for neoadjuvant chemotherapy. Patients who had received prior chemotherapy for OGC were excluded. The full inclusion and exclusion criteria are detailed in Supplementary Appendix A. All participants provided written informed consent.

### 2.2 Study design and treatment

For dose escalation, a rolling-six design [19] was used, with three oral AZD8931 dose levels: 20 mg bd, 40 mg bd, and 60 mg bd. Two intermittent schedules of AZD8931 were investigated once recruitment to the 20 mg bd group was complete: 14 days on/7 days off (schedule 1) and 4 days on/3 days off every week in a cycle (schedule 2). Patients would receive oral AZD8931 monotherapy twice daily for three days, then together with Xelox chemotherapy from day 4 for eight 21-day cycles (Xelox: oxaliplatin at 130 mg/m^2^ IV over 2 h on day one of every cycle; capecitabine 1250 mg/m^2^ bd for cycle duration). AZD8931 could continue after cessation of Xelox providing there was no evidence of tumour progression and treatment was tolerated.

The dose expansion phase was an open-label study, with patients randomised 2:1 to receive either Xelox + AZD8931 at AZD8931 RP2D for two 21-day cycles, or Xelox alone as neoadjuvant treatment. Patients without disease progression (as per RECIST 1.1) would undergo radical oesophagogastric surgical resection with extended lymph node dissection 4-6 weeks after completing neoadjuvant chemotherapy. Those who received AZD8931 in the expansion phase could commence maintenance AZD8931 at the same dose, 6–12 weeks after successful surgical resection (complete [R0]/marginal [R1]) for up to 12 months (if no evidence of recurrent disease). Safety follow-up was scheduled for four weeks after AZD8931 treatment ended. Tumour response assessment was performed via CT scan at 43 days from commencing neoadjuvant treatment to determine eligibility for surgery and at six months after surgery for analysis of outcomes.

### 2.3 Sample size

The maximum number of participants for dose escalation was 42: six patients taking one of three dose levels for two intermittent schedules, plus up to six patients recruited before implementation of the intermittent schedules.

Sample size for the expansion phase was based on observing outcomes that would render the AZD8931 regimen worthy of further investigation: with 20 patients receiving AZD8931, 78% progression-free survival (PFS) at 6 months from surgery corresponds to a lower one-sided 95% confidence limit for true 6-month PFS of 54%. In addition, an 80% R0 resection rate among 20 patients corresponds to a lower one-sided 80% confidence limit of 64%. Patients were randomised 2:1 with the majority receiving the treatment combination. One reason for this was to ensure sufficient numbers to evaluate feasibility of maintenance treatment, as drop-out after surgery was envisaged. The Xelox alone (reference) arm size was 10, giving a total of 30 patients to be randomised in the expansion phase.

### 2.4 Statistical analysis

The primary objective of the study was to determine the MTD of AZD8931 in combination with Xelox, defined as the highest dose level at which fewer than 2 of 6 patients experienced a dose limiting toxicity (DLT). DLTs were based on clinical and laboratory toxicity assessments (defined by National Cancer Institute-Common Toxicity Criteria, version 4.0, with the full definition provided in Supplementary Appendix B). Patients were evaluable for dose escalation analysis if they completed cycle 1 of AZD8931 treatment or withdrew from cycle 1 because of a DLT.

Secondary outcomes (dose expansion phase only) were PFS, PFS at 6 months, R0 rate at surgery, OS, OS at 12 months and safety. PFS was defined as time (days) from randomisation to progression (determined by RECIST 1.1) or death from any cause. Patients without disease progression and alive at the end of the study were censored at the date they were last known to be alive and progression-free. R0 rate was defined as the proportion of patients achieving a complete surgical resection divided by the total number of patients randomised in the respective arm. OS was defined as the time (days) from randomisation to death (any cause). Patients who were alive at the end of the study were censored at the date they were last known to be alive. Adverse events (AEs) were defined using the Common Terminology Criteria for Adverse Events, v4.03, and collected from the first day of treatment up to 30 days after ending treatment. Safety analyses were performed on an as-treated basis in both phases of the study. There were two safety populations in the expansion phase: patients who received at least one dose during the neoadjuvant period and patients who received at least one dose of maintenance AZD8931 postoperatively. Escalation phase safety data were summarised descriptively for each dose cohort separately and overall, with the number and percentage of patients experiencing each type of AE reported. Expansion phase safety data were summarised in accordance with the type and grade of AE, with number and percentage of patients experiencing the AE reported. Efficacy analyses (expansion phase only) were performed on an intention-to-treat basis. In the expansion phase, median follow-up time was calculated using the reverse Kaplan-Meier method [20]. The six-month PFS (90% CI), 12-month OS (90% CI) and 24-month OS (90% CI) estimates were taken from Kaplan-Meier survival curves. R0 rate was compared between groups using Fisher's exact test. Analyses were undertaken using Stata, version 15.1 (StataCorp, College Station, TX, USA) and R, version 3.4.2. A (two-sided) 10% significance level was used.

## 3 Results

### 3.1 Patients

**Fig. 1:**
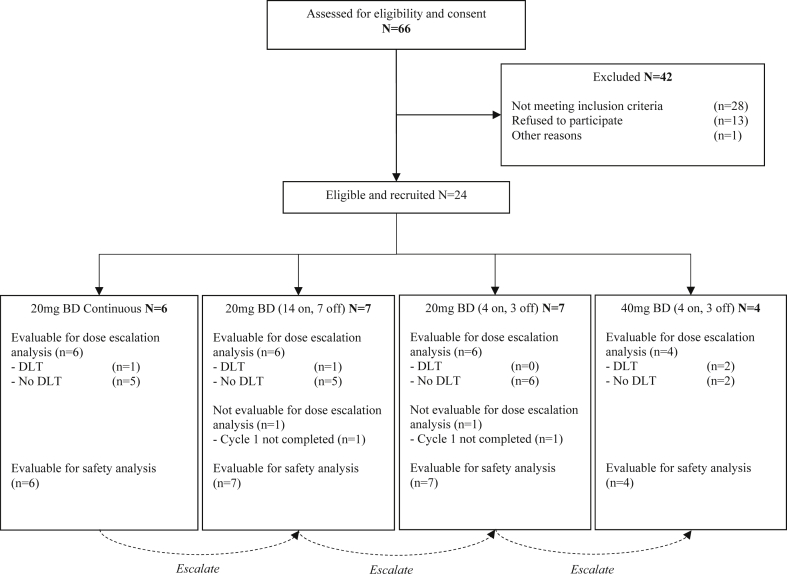
CONSORT diagram showing patient flow for the phase I dose escalating study component for AZD8931 in combination with oxaliplatin and capecitabine (Xelox) chemotherapy in patients with oesophago-gastric adenocarcinoma. BD, bi-daily.

**Fig. 2:**
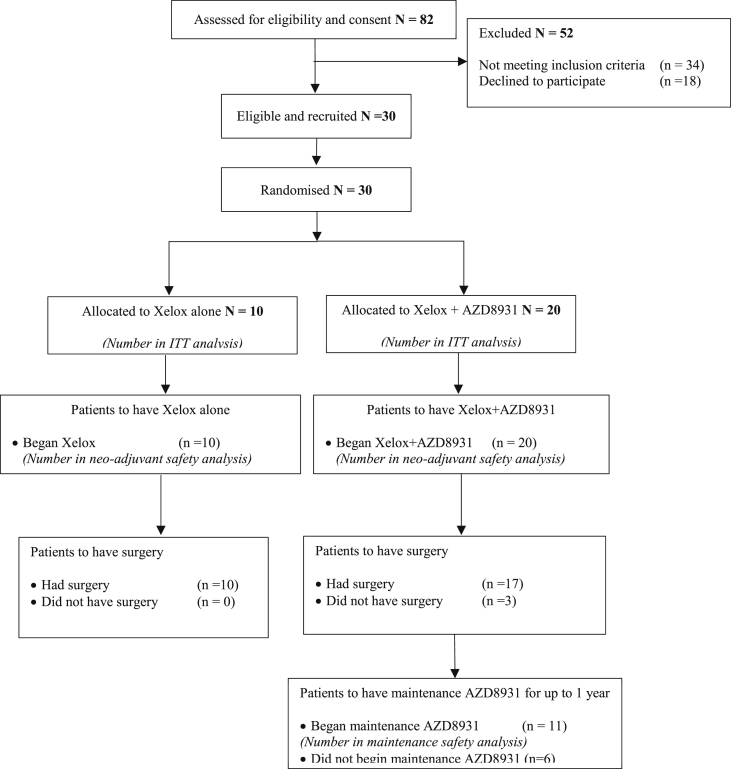
CONSORT diagram showing patient flow for the randomised phase II dose expansion study component for AZD8931 in combination with oxaliplatin and capecitabine (Xelox) chemotherapy in patients with oesophagogastric adenocarcinoma vs Xelox alone. ITT, intention to treat.

Twenty-four patients were recruited to the escalation phase between June 2012 and October 2014 in three ECMC UK centres (Oxford, Leicester and Belfast). Thirty patients were randomised to the expansion phase between March 2015 and May 2016 in five ECMC UK centres (Oxford, Leicester, Belfast, Bristol and Leeds) and follow-up ended in November 2017. Consolidated Standards of Reporting Trials (CONSORT) diagrams are shown for escalation and expansion phases (Fig. 1 and Fig. 2, respectively). Baseline demographics for escalation and expansion phases are summarised in Table 1, Table 2, respectively. The number of patients by HER2 status in addition to sample discordance between those collected at baseline (biopsy) and at surgery is described in Table 3.

**Table 1:** Baseline demographics for patients in the dose escalation phase (n = 24).

Variable	20 mg BD Cont. (n = 6)	20 mg BD 14on 7 off (n = 7)	20 mg BD 4on 3 off (n = 7)	40 mg BD 4on 3 off (n = 4)	Total (n = 24)
Age, in years, median (range)	63 (35–72)	74 (52–78)	60 (43–69)	54 (39–69)	62 (35–78)
Gender male, n (%)	5 (83%)	6 (86%)	4 (57%)	3 (75%)	18 (75%)
WHO PS 0, n (%)	5 (83%)	5 (71%)	6 (86%)	3 (75%)	19 (79%)
WHO PS 1, n (%)	1 (17%)	2 (29%)	1 (14%)	1 (25%)	5 (21%)
HER2 status positive, n (%)	3 (50%)	2 (29%)	2 (29%)	1 (25%)	8 (33%)
HER2 status negative, n (%)	3 (50%)	5 (71%)	4 (57%)	3 (75%)	15 (63%)
HER2 status unknown, n (%)	0	0	1 (14%)	0	1 (4%)
Locally advanced disease, n (%)	0	0	2 (29%)	0	2 (8%)
Metastatic disease, n (%)	6 (100%)	7 (100%)	5 (71%)	4 (100%)	22 (92%)
Prior radiotherapy, n (%)	0	1 (14%)	0	0	1 (4%)

BD, bi-daily; WHO PS, World Health Organisation performance status; HER2, human epidermal growth factor receptor-2.

**Table 2:** Baseline demographics for the expansion phase by treatment group (n = 30).

Variable	AZD893 + XELOX (n = 20)	XELOX (n = 10)	Total (n = 30)
Age, years, median (range)	63 (50–78)	66 (25–75)	64 (25–78)
Gender male, n (%)	17 (85%)	10 (100%)	27 (90%)
WHO PS 0, n (%)	17 (85%)	7 (70%)	24 (80%)
WHO PS 1, n (%)	3 (15%)	3 (30%)	6 (20%)
HER2 status, positive, n (%)	1 (5%)	0	1 (3%)
HER2 status, negative, n (%)	15 (75%)	8 (80%)	23 (77%)
HER2 status, unknown^a^, n (%)	4 (20%)	2 (20%)	6 (20%)
EGFR status, positive, n (%)	6 (30%)	4 (40%)	10 (33%)
EGFR status, negative, n (%)	10 (50%)	4 (40%)	14 (47%)
EGFR status, unknown^a^, n (%)	4 (20%)	2 (20%)	6 (20%)
Siewert type I, n (%)	4 (20%)	1 (10%)	5 (17%)
Siewert type II, n (%)	7 (35%)	2 (20%)	9 (30%)
Siewert type Oesophagus, n (%)	9 (45%)	7 (70%)	16 (53%)

EGFR, epidermal growth factor receptor; HER2, human epidermal growth factor receptor-2; WHO PS, World Health Organisation performance status.

a Status unknown at biopsy because of block being unavailable (5); did not consent for use (1).

**Table 3:** Number of patients by HER2 status. Discordance in HER2 status is shown between diagnostic biopsy and resection specimens.

Diagnostic Biopsy	Resection specimens					
	Xelox + AZD8931 (n = 20)			Xelox (n = 10)		
HER2 status	Positive	Negative	Unknown	Positive	Negative	Unknown
Positive	0	1	0	0	0	0
Negative	2	9	4	0	6	2
Unknown	0	1	3	1	0	1

### 3.2 Treatment compliance

In the escalation phase, all patients received AZD8931 monotherapy in the 3-day run in period without missed or reduced doses. In cycle 1 of AZD combined with Xelox, five patients missed AZD8931 doses because of AEs (fatigue, diarrhoea, poor oral, vomiting and coronary spasm), by own choice, or in error. Three patients did not complete cycle 1 of Xelox: one withdrew consent, one because of experiencing a rash, and one because of experiencing a coronary spasm. Over all cycles, patients stopped AZD8931 because of toxicity (n = 7, 29%), disease progression (n = 16, 67%) or consent withdrawal (n = 1, 4%).

In the expansion phase, 14 of 20 patients (70%) completed two cycles of neoadjuvant AZD8931 treatment in the Xelox + AZD8931 group. Three (15%) did not complete two cycles: one due to an AE (out of range liver function test) and two due to a serious adverse event (SAE) (diarrhoea). AZD8931 diary cards for the other three patients were unavailable, so it could not be confirmed that they completed the two cycles of AZD8931. Seventeen patients (85%) completed two cycles of neoadjuvant Xelox treatment; all patients received their allocated oxaliplatin, but two patients did not complete capecitabine treatment due to a SAE (diarrhoea). The diary card for one patient was unavailable, so it could not be confirmed whether the two cycles of capecitabine were completed.

Of the 10 patients in the Xelox-alone group, six (60%) completed two cycles of neoadjuvant Xelox. Two patients (20%) did not complete two cycles because of a SAE (diarrhoea) and AE (atrial fibrillation). Diary cards for the remaining two patients were unavailable, so it could not be confirmed whether they completed the two cycles of AZD8931.

In the Xelox+AZD8931 group, 17 of 20 patients had surgery, compared with 10 of 10 patients in the Xelox-alone group. Of the 17 patients in the Xelox + AZD8931 group who had surgery, 11 continued to AZD8931 maintenance, with six completing 12 months of treatment. Three patients stopped treatment because of disease progression, one as per the decision of the treating clinician and one in error after taking approximately 11 months of maintenance AZ8931.

### 3.3 Adverse events

During dose escalation, there were 428 grade I–IV AEs: 77 occurred during cycle 1 and 351 after cycle 1. All but one patient experienced at least four AEs during the escalation phase. There were 62 grade III–IV AEs: six during cycle 1 and 56 after cycle 1. AEs at grade III were experienced by 20 (83%) patients, with the most common grade III–IV AEs being diarrhoea (n = 7, 29%) and vomiting (n = 4, 17%) (Table 4). Thirty-three SAEs in fifteen (63%) patients were reported during dose escalation: four during cycle 1 and 29 after cycle 1. Of these SAEs, 39% (13/33) were deemed related to AZD8931. Common SAEs were diarrhoea (n = 9, 38%) and vomiting (n = 4, 17%). There were six suspected unexpected serious adverse reactions (SUSARs) among four patients, none of which were deemed definitely related to AZD8931: three (13%) patients experienced vomiting (one at grade II; two at grade III; all SAEs were possibly related to AZD8931) and two (8%) experienced diarrhoea (all at grade III and possibly or probably related to AZD8931). Note: one patient had both diarrhoea (on two separate occasions) and vomiting.

**Table 4:** Number (%) of patients with grade III–IV AEs of each type during the escalation phase^a^.

n (%)	20 mg Continuous (n = 6)	20 mg 14ON 7OFF (n = 7)	20 mg 4ON 3OFF (n = 7)	40 mg 4ON 3OFF (n = 4)	Total (n = 24)
Diarrhoea	3 (50%)	1 (14%)	1 (14%)	2 (50%)	7 (29%)
Vomiting	–	3 (43%)	1 (14%)	–	4 (17%)
Nausea	1 (17%)	–	1 (14%)	–	2 (8%)
Gastrointestinal haemorrhage	2 (33%)	–	–	–	2 (8%)
Neutropenia	–	2 (29%)	–	–	2 (8%)
Pyrexia	–	1 (14%)	–	1 (25%)	2 (8%)
Fatigue	2 (33%)	–	–	–	2 (8%)
Dehydration	1 (17%)	–	1 (14%)	–	2 (8%)
Pulmonary embolism	1 (17%)	1 (14%)	–	–	2 (8%)

a Only AEs occurring in ≥5% of patients are reported in this table.

**Fig. 3:**
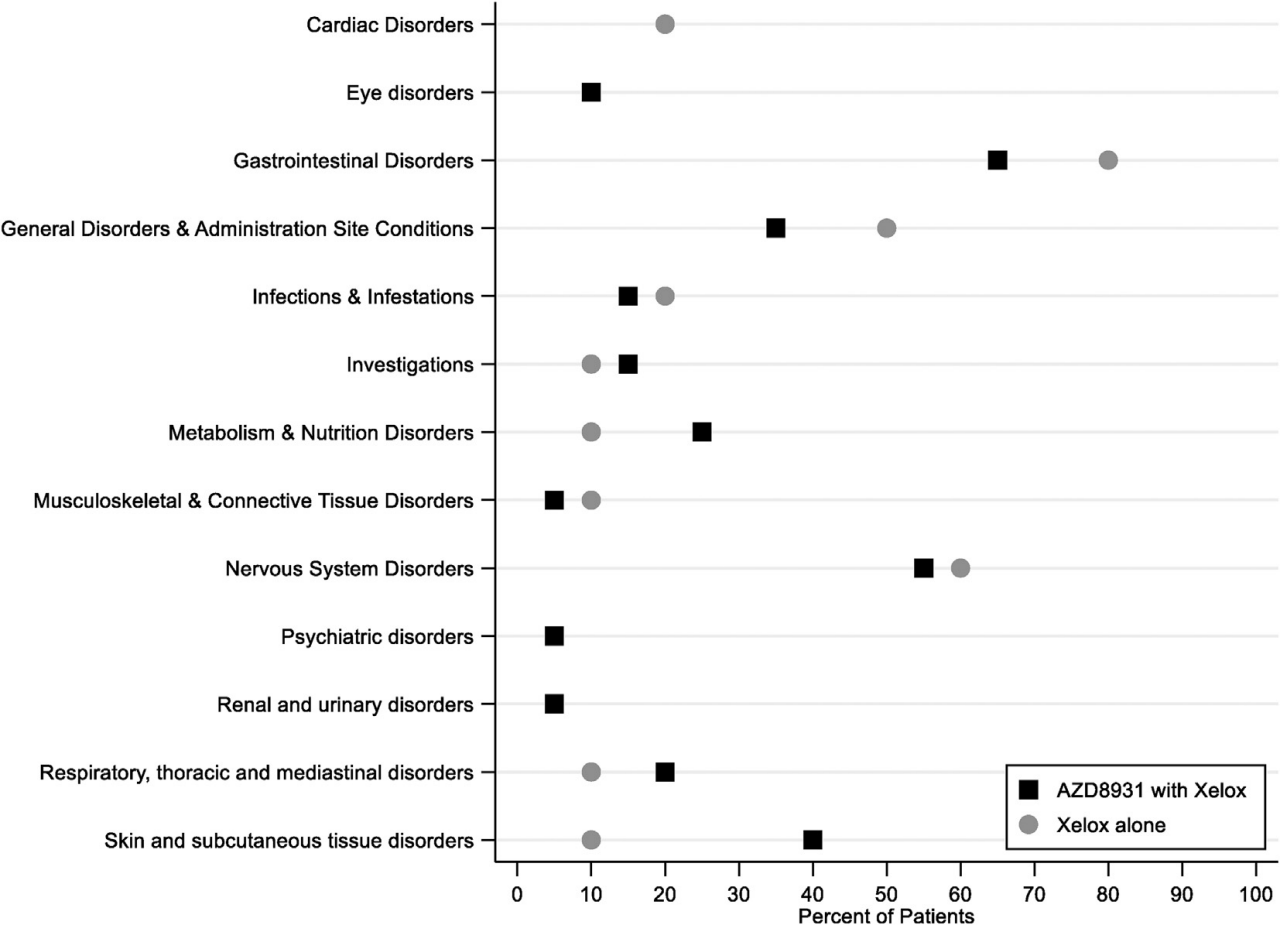
Percentage of patients with AEs in each system organ during neoadjuvant treatment in the expansion phase.

During neoadjuvant treatment in the expansion phase, 144 AEs were reported in total. In the Xelox-alone group, 90% (9/10) of patients had an AE, compared with 80% (16/20) in the Xelox + AZD8931 group. The most common AEs were diarrhoea (9/20, 45% Xelox + AZD8931; 4/10, 40% Xelox alone), nausea (8/20, 40% Xelox + AZD8931; 2/10, 20% Xelox alone) and fatigue (7/20, 35% Xelox + AZD8931; 5/10, 50% Xelox alone). AEs by system organ are reported in Fig. 3. Grade III–IV AEs were reported in 2 of 20 (10%) patients in the Xelox + AZD8931 group and 5 of 10 (50%) patients in the Xelox-alone group, with the most common being diarrhoea and vomiting (Table 5). Grade III–IV diarrhoea was deemed related to Xelox treatment for both patients in the Xelox + AZD8931 group and related to AZD8931 for one of them. In the Xelox only group, grade III–IV diarrhoea was related to the Xelox treatment for one patient. There were 13 SAEs during the neoadjuvant treatment period, with 43% (3/7) reported among the Xelox + AZD8931 group deemed related to AZD8931. The most common SAE was diarrhoea, of which there were four occurrences (Table 6).

**Table 5:** Number (%) of patients with grade III-IV adverse events (AE) of each type during neoadjuvant treatment for the randomised expansion phase.

AE term	Xelox + AZD8931 (n = 20)	XELOX (n = 10)
Diarrhoea^a^	2 (10%)	2 (20%)
Vomiting^a^^,^^b^	1 (5%)	1 (10%)
Hypophosphataemia	–	1 (10%)
Liver function test abnormal^b^	–	1 (10%)
Pulmonary embolism^a^	1 (5%)	1 (10%)
Sepsis	–	1 (5%)

a One patient in the AZD893 + XELOX group had three grade III–IV AEs: diarrhoea, vomiting and pulmonary embolism.

b One patient in the XELOX-alone group had two grade III–IV AEs: vomiting and liver test function abnormal.

**Table 6:** Serious adverse events (SAEs) that occurred during neoadjuvant treatment for the expansion phase, by treatment group.

Treatment group	SAE	Grade	Related to		
			AZD8931	Capecitabine	Oxaliplatin
Xelox + AZD8931	Diarrhoea	II	Probably	Probably	Probably
Xelox + AZD8931	Diarrhoea	111	Possibly	Possibly	Probably not
Xelox + AZD8931	Diarrhoea	111	Possibly	Definitely	Possibly
Xelox + AZD8931	Haematuria	I	Probably not	Definitely not	Definitely not
Xelox + AZD8931	Haematuria	I	Probably not	Definitely not	Definitely not
Xelox + AZD8931	Pain	I	Probably not	Probably not	Probably not
Xelox + AZD8931	Vomiting	III	Definitely not	Probably	Possibly
Xelox	Diarrhoea	III	NA	Definitely	Possibly
Xelox	Dyspepsia	II	NA	Possibly	Probably
Xelox	Hypophosphatemia^a^	IV	NA	Probably not	Probably
Xelox	Out of range LFTs	III	NA	Probably	Possibly
Xelox	Sepsis	III	NA	Definitely	Definitely
Xelox	Vomiting	III	NA	Probably	Possibly

LFT, liver function test; NA, not applicable.

a SUSAR.

All 11 patients experienced an AE during AZD8931 postoperative maintenance. In total, there were 33 AEs (n = 19, 58% related to AZD8931), with the most common grade I–IV AE being skin rash, experienced by four patients (36%) on AZD8931 maintenance (related to AZD8931 in three patients). There were two grade III AEs: metastases to central nervous system and arthritis, both considered not to be related to AZD8931. One SAE reported during maintenance (grade III brain metastasis) was considered related to the underlying disease.

Two SUSARs were reported during the expansion phase: thoracotomy wound dehiscence (Xelox + AZD893) and hypophosphataemia (Xelox-only), both of which were grade IV.

### 3.4 Maximum tolerated dose

In total, 22 of 24 patients were evaluable for the dose escalation analysis. Four DLTs were observed amongst 22 patients: (1) failure to deliver 100% of the planned dose of Xelox because of grade III fatigue attributable to AZD8931 with or without Xelox; (2) failure to deliver 100% of the planned dose of Xelox because of grade III diarrhoea and vomiting attributable to AZD8931 with or without Xelox; (3) failure to deliver 100% of the planned dose of Xelox because of grade III vomiting attributable to AZD8931 with or without Xelox; (4) grade III rash which persisted for at least 5 days despite optimal treatment. No DLTs were observed in the 20-mg bd 14d on/3d off schedule, which was declared as the RP2D.

### 3.5 Survival and resection rates

**Fig. 4:**
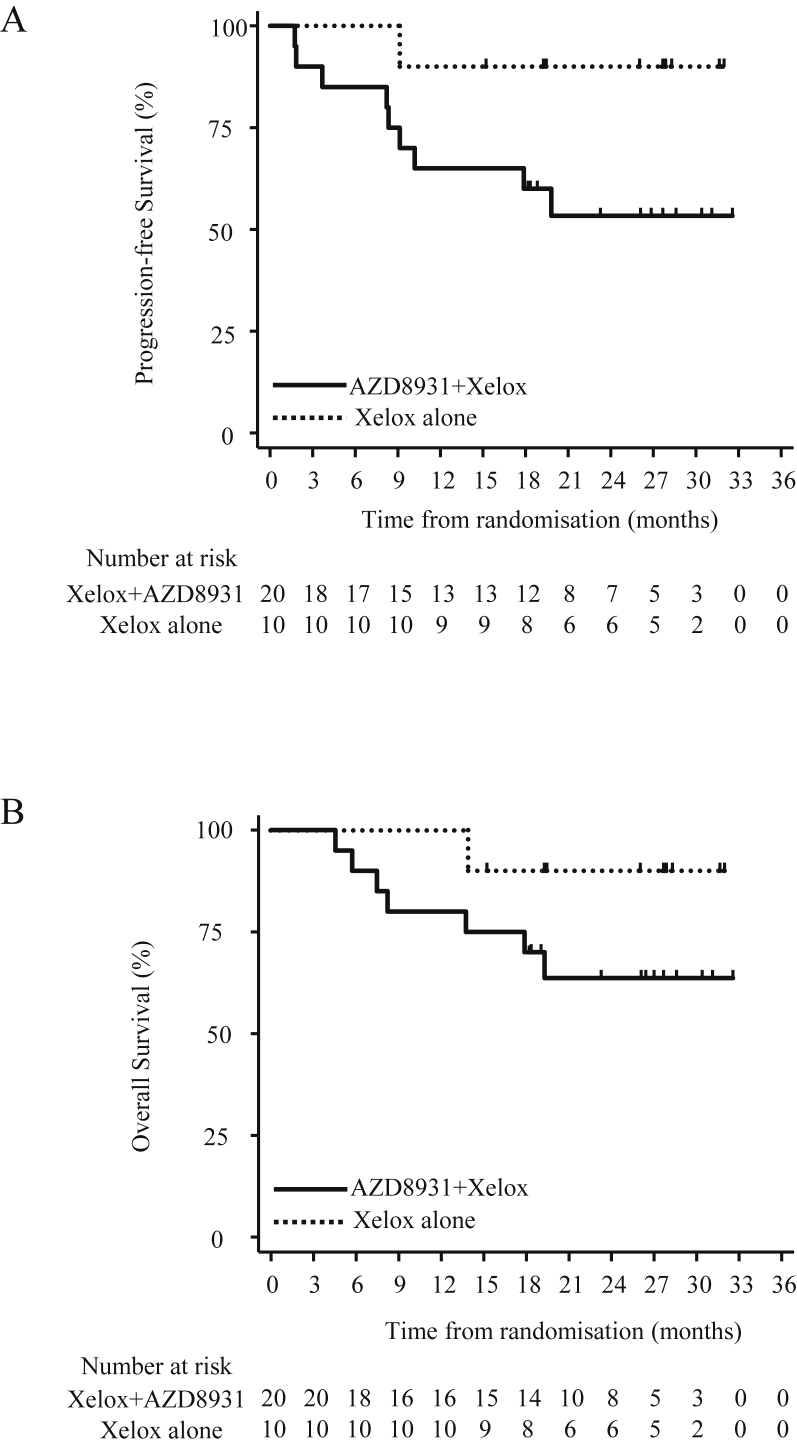
Kaplan-Meier plots showing progression-free survival as per RECIST 1.1 (A) and overall survival (B) for participants receiving AZD8931 + Xelox vs Xelox alone in the expansion phase.

In the dose expansion phase, median follow-up was 26.8 months. Ten patients (33%) progressed or died during the course of the expansion phase: 9 (45%) patients in the Xelox + AZD8931 group and 1 (10%) in the Xelox-alone group. Median PFS could not be estimated in either groups (Fig. 4A). The lower 90% confidence limit for median PFS in the Xelox + AZD8931 group was estimated as 9.1 months. PFS at six months was 85% (90% CI: 66%, 94%) in the Xelox + AZD8931 group and 100% in the Xelox-alone group.

Eight deaths (all disease-related) occurred during the expansion phase; 7 (35%) deaths in the Xelox + AZD8931 group and 1 (10%) in the Xelox-alone group. OS at 12 months was 87% (90% CI: 72%, 94%) overall: 80% (90% CI: 60%, 91%) in the Xelox + AZD8931 group and 100% in the Xelox alone group (Fig. 4B). OS at 24 months was 72% (90% CI: 56%, 84%) overall: 64% (90% CI: 43%, 79%) in the Xelox + AZD8931 group and 90% (90% CI: 58%, 98%) in the Xelox-alone group. Median OS time could not be estimated for either group. The lower 90% confidence limit for median OS time in the Xelox + AZD8931 group was estimated as 17.9 months.

The proportion of patients achieving R0 resection at surgery was 45% (n = 9) in the Xelox + AZD8931 group and 90% (n = 9) in the Xelox-alone group (*P* = 0.024). All patients who underwent surgery achieved either R0 or R1 resection.

## 4 Discussion

Defining new treatment options in OGC is critical for improving outcomes. Approximately 30% of patients with OGC have disease characterised by HER2 amplification [21] and benefit from treatment with the anti-HER2 monoclonal antibody, trastuzumab. However, benefit may also be realised in HER2-low or HER2-negative tumours by targeting the wider EGFR family. ErbB3 expression, for example, has been increasingly characterised in oesophageal cancers [22,23] and is associated with poor prognosis [24]. Furthermore, erbB3 plays a central role in signal transduction via the phosphatidylinositol-3-kinase pathway, activating both EGFR and HER2 to further deregulate pro-proliferative signalling networks. AZD8931 is an equipotent reversible inhibitor of EGFR, erbB2, and erbB3 [25] which may therefore offer added therapeutic benefit across a wider range of OGC molecular subtypes.

The primary end-point for DEBIOC was to determine the MTD for AZD8931 in combination with Xelox chemotherapy in patients with OGC. The four DLTs observed in the escalation phase included the AEs diarrhoea and vomiting, reflecting the most commonly reported AEs across all dose levels. A dose finding study of AZD8931 in patients with advanced solid tumours by Tjulandin *et al.* [26], gave bi-daily single-agent dosing from 40 to 300 mg. Here, diarrhoea was also the most common AE across all doses and contributed to two DLTs in the 300-mg cohort. However, in FOCUS-4, a molecularly stratified randomised trial in patients with colorectal cancer, a 40 mg–20 mg dose reduction in AZD8931 was mandated primarily because of skin rash in 20% of patients [27]. The multi-institutional, neoadjuvant therapy (MINT) study assessed the combination of AZD8931 with anastrozole in breast cancer patients, revealing an increased incidence of diarrhoea, rash, and acneform dermatitis compared with placebo [28]. In addition, discontinuation of anastrozole was reported at greater rates for those receiving AZD8931 than placebo. In contrast, during the expansion phase of DEBIOC, diarrhoea was reported at similar rates for both arms whereas overall grade III–IV AEs were reported in 10% patients in the Xelox + AZD8931 group compared with 50% patients receiving Xelox alone, suggesting that this combination is both safe and tolerable. DEBIOC is also the first study to consider AZD8931 in long-term postsurgical maintenance therapy, during which time 58% patients experienced AZD8931-related AEs, the most common being skin rash. Although skin rash is a common grade III–IV toxicity typically occurring in 10–20% of patients receiving tyrosine kinase inhibitors [29], no events of this nature ≥ grade III were observed with AZD8931 in the expansion phase.

The discordance between the diagnostic biopsy and resection specimens for both HER2 and EGFR status, demonstrates potential heterogeneity of expression in these cancers or indeed a neoadjuvant treatment effect. There is clear evidence to support molecular stratification to identify those patients who will gain clinical benefit from being exposed to targeted agents [13,30]. Eliminating this discordance is essential if we are going to accurately stratify patients to receive targeted agents.

Neoadjuvant chemotherapy offers significant survival benefit (equating to approximately 7% at 2 years) in OGC compared with surgery alone [31,32]. Previous studies specifically assessing neoadjuvant Xelox in oesophageal cancer estimated a 2-year OS to be 42% and PFS to be 32.5% [33]. In the UK MRC OE05 study, OS at two years was approximately 50% (taken from their Kaplan-Meier curve [10]. In DEBIOC, OS at two years was 72% (90% CI: 56%, 84%). In DEBIOC, median PFS in both arms was not established because of the small proportion of events per group. R0 resection rates of 90% in the Xelox-only group were significantly better than for the AZD8931 arm but were also much higher than would be expected for Xelox alone, with R0 resection following neoadjuvant chemotherapy typically ranging from approximately 59%–82% [5,10,34,35]. The small size of this study is likely a major contributing factor to these disparities.

## 5 Conclusions

The RP2D of the equipotent inhibitor of EGFR, erbB2, and erbB3, AZD8931, in combination with standard-of-care neoadjuvant Xelox chemotherapy in resectable patients with OGC is 20-mg bd (4 days on/3 off every week). Although the sample size was too small to draw conclusions regarding efficacy, this study shows that expansion of triplet neoadjuvant therapy to include a pan-erbB inhibitor, where specific HER2-targeting therapies may not be appropriate, appears both safe and tolerable.

## Funding

This work was supported by AstraZeneca, Cancer Research UK [C10604/A14112] the Experimental Cancer Medicine Centre (ECMC) and NIHR Clinical Research Network [UKCRN ID 11855]. Additional NHS clinical service support costs for patient care while on study were met by the hosting sites. This study was part of the NIHR portfolio.

## Conflict of interest statement

A.T., M.E., S.R.L., S.F., D.A.A., R.C.T., M.G., L.E., and S.L. declare no conflict of interest. During the conduct of the study, P.S.V. and L.C. report receiving grants from AstraZeneca. J.M. reports grants from AstraZeneca and Cancer Research UK. M.R.M. reports grants from Roche, AstraZeneca and GSK; received personal fees from Amgen, Roche, GSK, Novartis, Immunocore, BMS, Eisai, Merck, Rigontec, BiolineRx and Array Biopharma; received non-financial support from Immunocore and Merck; has been a member of the Advisory Board/and has also received study fees (institution only) from Novartis, Millennium, Immunocore, BMS, Vertex, Eisai, Pfizer, Merck, Rigontec, Regeneron, TCBiopharma, Array Biopharma and Replimune; and also having IDSMC membership with Eisai.

## Appendix A Supplementary data

Supplementary Appendix A Full study inclusion and exclusion criteria Supplementary Appendix A

Supplementary Appendix B Dose Limiting Toxicity Criteria for the escalation phase Supplementary Appendix B
