# Supplementary material for: Dual Erb B Inhibition in Oesophago-gastric Cancer (DEBIOC): A phase I dose escalating safety study and randomised dose expansion of AZD8931 in combination with oxaliplatin and capecitabine chemotherapy in patients with oesophagogastric adenocarcinoma
Source: Eur J Cancer. 2020 Jan;124:131–41. doi: 10.1016/j.ejca.2019.10.010 (PMC6947485; doi:10.1016/j.ejca.2019.10.010)
Supplement: Supplementary Appendix A — Full study inclusion and exclusion criteria [file mmc1.docx]

Supplementary Appendix 1.

**Inclusion Criteria: All patients**

- Age ≥ 18 years
- WHO performance status 0-1
- Adequate respiratory and cardiac function
- Able to give informed consent and be capable of co-operating with protocol
- Haematological and biochemical indices within the ranges shown below:
- Haemoglobin (Hb) ≥10g/dl,
- Neutrophils≥ 2000/µl,
- Platelet count ≥ 100.000/µl,
- AST or ALT ≤ 3 ULN, alkaline phosphatase ≤ 2x ULN,
- Serum Bilirubin ≤ 1.5 ULN,
- Creatinine Clearance ≥ 50ml/min (Calculated by Cockcroft Gault equation, or by EDTA)
- Able to swallow oral medication
- Male patients must use a barrier method of contraception (female condom or diaphragm are not acceptable) during the study and after cessation of therapy for 6 months.

**Inclusion Criteria: Dose escalation phase only**

- Patients with locally advanced or metastatic gastro-oesophageal adenocarcinoma, i.e. inoperable
- Women of child bearing potential must use an acceptable method of contraception during the study and after cessation of therapy for 4 months, and have a negative pregnancy test

**Inclusion Criteria: Dose expansion phase only**

- Histologically confirmed carcinoma of the oesophagus and gastro-oesophageal junction [GOJ] Siewert Type I and II
- Operable disease: any combination T1-3 / N0-1 [BUT EXCLUDES T1N0]; T4 involvement of mediastinal pleura and diaphragmatic crus where the MDT consider this resectable
- Deemed suitable for neo-adjuvant chemotherapy by regional upper GI MDT
- Women Not of Childbearing Potential i.e. women who are postmenopausal or permanently sterilised (e.g. tubal occlusion, hysterectomy, bilateral salpingectomy). Pregnancy test must be done for women who have been post-menopausal for less than 2 years.

**Inclusion Criteria: Dose Maintenance phase**

- Patients who have successful surgery
- Patients who received AZD8931 treatment in the expansion phase

**Exclusion Criteria: All patients**

- Previous chemotherapy for oesophago-gastric adenocarcinoma
- Squamous cell pathology
- Uncontrolled angina, myocardial infarction within 6 months, heart failure or impaired LV function on echocardiogram/MUGA, uncontrolled arrhythmias
- History of interstitial lung disease
- Known peripheral neuropathy >Grade 1
- Other experimental treatment ≤ 4 weeks prior to this study (including chemotherapy and immunotherapy)
- Known or expected dihydropyridime dehydrogenase deficiency
- Resting ECG with QTc >480msec at 2 or more time points within a 24h period
- Requirement for medication known to inhibit or induce CYP3A4 or 2D6, or medication known to prolong QT interval.
- History of other malignancy less than 5 years before the diagnosis of oesophageal cancer, EXCLUDING the following: Non-melanoma skin cancer, in situ carcinoma of the cervix treated surgically with curative intent, other malignant tumours that have been treated curatively and patient is deemed disease-free
- Active infections (including chronic hepatitis type B or C and HIV infection if status known), severe immunologic defect, compromised bone marrow function
- Prior diagnosis of dry eye syndrome or eye-lid/eye-lash abnormalities.  History of eye injury, corneal surgery, orbital irradiation, collagen vascular, chronic inflammatory or denegerative disease with eye involvement, clinically significant ocular surface disease
- Known hypersensitivity to any component of chemotherapy
- Pregnancy, inadequate or unreliable contraceptive measures during participation in the trial; breast feeding.
- Other psychological, social or medical condition, physical examination finding or a laboratory abnormality that the Investigator considers would make the patient a poor trial candidate or could interfere with protocol compliance or the interpretation of trial results.

**Exclusion Criteria: Dose expansion phase and dose maintenance phase only**

- Siewert Type III GOJ tumours and gastric cancer
- Women of child bearing potential i.e. Any female who has experienced menarche and does not meet the criteria for “Women Not of Childbearing Potential”
