# Supplementary material for: Dual Erb B Inhibition in Oesophago-gastric Cancer (DEBIOC): A phase I dose escalating safety study and randomised dose expansion of AZD8931 in combination with oxaliplatin and capecitabine chemotherapy in patients with oesophagogastric adenocarcinoma
Source: Eur J Cancer. 2020 Jan;124:131–41. doi: 10.1016/j.ejca.2019.10.010 (PMC6947485; doi:10.1016/j.ejca.2019.10.010)
Supplement: Supplementary Appendix B — Dose Limiting Toxicity Criteria for the escalation phase [file mmc2.docx]

Supplementary Appendix 2.

**Dose Limiting Toxicities**

- Grade III/IV neutropenia with sepsis or with fever > 38.5 °C
- Grade IV thrombocytopenia
- Grade IV diarrhoea
- Symptomatic ophthalmic toxicity (epitheliopathy or erosion) which does not recover within 3 days of detection
- Grade ≥ III rash which persists for 5 days or more despite optimal treatment
- QTc prolongation > 500 msec or QTc increase > 60 msec above screening value
- Grade ≥ III other non-haematological toxicities except for alopecia, and nausea or vomiting unless patients are taking optimal prophylaxis or supportive measures
- Delay of > 2 weeks in administration of cycle 2 of treatment due to haematological or non-haematological toxicities
- Failure to deliver 100% of the planned dose of Xelox due to toxicity attributable to AZD8931 or the AZD8931/Xelox combination
